# Supplementary material for: Detection of Cancer Recurrence Using Systemic Inflammatory Markers and Machine Learning after Concurrent Chemoradiotherapy for Head and Neck Cancers
Source: Cancers (Basel). 2023 Jul 8;15(14):3540. doi: 10.3390/cancers15143540 (PMC10377662; doi:10.3390/cancers15143540)
Supplement: Supplementary file 1 [file cancers-15-03540-s001.zip › cancers-2457231-supplementary/Supple_Table S1.pdf]

Table S1

External validation dataset.

Characteristics		Values
Age (yr)	Mean ± SD	59.4 ± 12.0
Gender (Male/Female)		136/37
Smoking		116 (67.1%)
Stage*	I	7 (4.0 %)
	II	24 (13.9 %)
	III	48 (27.7 %)
	IV	94 (54.3 %)
Tumor sites	Oral cavity	18 (10.4%)
	Larynx	8 (4.6%)
	Oropharynx	55 (31.8%)
	Hypopharynx	38 (22.0%)
	Nasopharynx	48 (27.7%)
	PNS/Nasal cavity	6 (3.5%)
Pathology	SCC	142 (82.1%)
	UDC	29 (16.8%)
	Others	2 (1.1%)
Recurrences		73 (42.2%)
RFS (*mo*)	Median (SE, 95%CI)	79.5 (26.2, 28.2~130.83)
5yr-RFS rate		51.7%

* Staging according to AJCC 7th edition. The value for one case was missing.

SD, standard deviation; SCC, squamous cell carcinoma; UDC, undifferentiated carcinoma; RFS, relapse-free survival; SE, standard error.
